# Redefining “Natural” in Agriculture

**DOI:** 10.1371/journal.pbio.0060199

**Published:** 2008-08-19

**Authors:** Tony Trewavas

## Abstract

Can organic farming and genetically engineered crops coexist in an agriculture of the future? Tony Trewavas reviews the new book*Tomorrow's Table*.

The place of genetically modified crops in sustainable agriculture has been the subject of heated debate for decades. A new book takes an innovative approach to this debate by presenting the perspectives of an unlikely pair of co-authors [1]. Pam Ronald is a plant molecular biologist, genetic engineer, and supporter of genetically engineering crops for the benefit of humanity. Raoul Adamchak is an organic farmer. Given the known antagonism of many organic advocates to genetically engineered (GE) crops, one would not have thought these two authors would be able to provide an agreed text. But Adamchak is married to Ronald and, to judge from the text, happily so. The authorship of the individual chapters alternates between the two. The subject matter deals with organic farming methods, GE methods, questions of environmental conservation, risk, trust, and ownership of seeds and genes. The last chapter, and the only one written jointly, concludes that some marriage of organic and GE technology will represent the agriculture of the future.

I must admit to holding the same view some 15 years ago, but not now. I assumed that the use of GE technology would be rather like the green revolution. Universities and research institutes would make new crop plants available and free to those that needed them. What has intervened of course for GE is the input of commercialism, which has muddied the waters. Organic farming is not immune to commercial pressures either, and there are strong suspicions that the organic industry's antagonism to GE is a marketing ploy. Mutated crops, induced by radiation, for example, have been used for many years by conventional and organic farmers alike, and it is now known that radiation mutation causes much greater genomic change than GE technology [2].

The text deals with many of the questions raised by the public about GE crops in a sensible and balanced manner, quoting various sources of reliable information on the concerns about risks to health and environment that often recur. It also mentions Richard Jefferson, who is Chairman of CAMBIA, a non-profit organisation that attempts to make the tools of biotechnology widely and freely available (http://www.cambia.org/). As a scientist, I cannot help but applaud!

A substantial part of the book gives accounts of conversations between Pam Ronald and others about GE, enabling her to easily justify why it is supported by many in universities. I particularly enjoyed an account of one conversation between Pam Ronald and a relative (a lawyer) who argued against GE crops. This particular discussion started because several counties of California had voted on an ordinance that would have banned GE farming. The relative had voted for the ordinance. I quote several passages to give a flavour to this discussion.

Lawyer: “I voted for the ordinance because it will send a message to the large corporations that the onus is on them to prove their products are safe.”

Ronald: “I point out that the ordinance contained no language concerning the role of corporations and I mention that the US National Academy of Sciences and the Royal Society in London (the supreme scientific bodies in these countries) have both indicated that these crops are safe.”

Lawyer: “Even if they are safe to eat I don't like the idea that many of the GE crops grown in the US are sprayed with herbicides.” [The reference here is to glyphosate, used to control weeds in GE herbicide-tolerant crops.]

Ronald: “The good thing about glyphosate is that it is known to be non-toxic to mammals and does not accumulate in water or in soil.”

Lawyer: “But even if the herbicide is non-toxic, I have read that there is a chemical mixed with the herbicide that can harm fish.” [This refers to a surfactant used in some glyphosate formulations.]

Ronald: “Well if it is the surfactant you object to, wouldn't it make more sense to simply ban the surfactant or the herbicide itself?”

Lawyer: “ It would be a political dead end to ban the herbicide because lots of people like to use it in their garden.”

The continuing conversation did not resolve the issues between them. It convinced me, however (if I needed convincing), that while everyone is entitled to their opinions, when dealing with detailed technical matters of science or medicine or any subject that requires enormous qualifications and experience, the notion that all opinions have equal validity is simply downright wrong. If you want real information on the safety of heart surgery procedures, do you follow the advice of a qualified heart surgeon or the local butcher? If you want advice on flying a jumbo jet, do you ask the local bus driver or a pilot with 10,000 hours of experience flying jumbo jets? And if you want advice on how to captain a supertanker, do you ask a person whose experience is limited to rowing a dinghy? Mistakes by surgeons are not uncommon, 70% of air crashes result from pilot error, and occasionally supertankers hit the rocks. But relying on rank amateurs instead of professionals would guarantee instant catastrophe. Many branches of science are very complex. However, being a scientist isn't enough, of course, as being a scientist doesn't qualify you to advise on any subject except your specialty. To provide advice that can lead to sensible policy requires not only a thorough understanding of the workings and literature of the particular scientific area but many decades of experience in that field.

It is unfortunate that for the past 40 years, agriculture in particular has been damaged by opinionated groups of the public that have forcefully used fear and anxiety and carefully selected information to try and coerce policy makers to adopt their own mistaken and unqualified views. Fear and emotion do not make for good policy. I applaud Ronald's conclusion that “if citizens vote, it should be for a specific matter on which they are well informed, not because of general concerns about a new technology.”

The corollary is that on most technical matters, the public can never be well enough informed. If scientific knowledge does not form the basis of policy on technology, basing such policy on ignorance can be guaranteed to generate disaster. It was Slovik in his classic *Perception of Risk* [3] who demonstrated that non-experts overestimate the frequency of death from rare causes while underestimating the frequency of common causes of death, and who established clearly how additional knowledge changed expert understanding. The use of the local ordinance by activist groups to stop GE farming is only too reminiscent of the damage done by Lysenkoism to Soviet farming in the 40s, which took decades to recover from, once it was abandoned.

Adamchak describes organic farming as working through biology and conventional farming as working through chemistry. The commonest reason the public gives for buying organic food is that it is natural (or biological; in France, organic food is described as *biologique*) and lacks “chemicals” because pesticides are not used to control pests. Adamchak's supposition is completely erroneous; plants have always used chemicals to control pests. Allelopathic plants kill other competing plants by secreting toxins from leaves and roots, and there are at least 100,000 natural toxins (pesticides) synthesised by higher plants to kill insect herbivores and found in all fruits and vegetables. When tested toxicologically on rodents, the daily average diet is known to contain natural pesticides that are nerve toxins, many carcinogens, endocrine disruptors (that either mimic oestrogen or induce male sterility), and other pesticides that interfere with cell division, cause chromosome breakage, or damage blood, skin, or thyroid. The list is remarkably similar to the claims made by activist groups about the biological effects of synthetic pesticides.

We are not adapted to exposure to these natural chemicals. The dozen or so nerve toxins in potato have in the past killed at least 30 people and caused sickness in thousands [4]; another nerve toxin, cucurbitacin in organic courgettes, has caused illness in large numbers of people. The endocrine disruptors (genistein and others) in soy are actually recommended to menopausal women to mitigate the symptoms arising from lower oestrogen content, while psoralen in parsnip, figs, and celery causes damaging skin blisters, and so on.

Natural pesticides represent about 1%–5% dry weight of any vegetable or fruit. There is a simple seesaw relation between yield and natural pesticide content. In crop breeding, the natural pesticide content has been reduced to increase yield, making produce safer for human consumption but now requiring additional synthetic pesticide to offset the reduced resistance. The margin of health safety for natural pesticides is small; for the traces of synthetic pesticides, it is enormous. If organic advocates object to the fact that traces of synthetic pesticides can be detected weeks after consumption, solanidine, one of the potato nerve toxins, has been shown to have a half life in the liver of several months. With present technology, the solanidine consumed in one potato could be detected years later, but this is almost the only natural pesticide whose biological stability has been examined. There is nothing unnatural about farmers using pesticides; biology uses chemistry to control pests. Natural certainly does not mean safe.

Although I could offer many other criticisms of individual parts of the book, I greatly enjoyed reading it. The most important omission that cannot be glossed over, however, is that of agricultural context. Support for any kind of agricultural technology depends simply on the context of the times. There is an old saying: “one food, one problem, much food, many problems.” For those whose next meal—and the next and the next—is a bowl of rice, other problems, such as whether food is organic or not, are of little consequence. Their one problem is where to get their next meal, and the only method of acceptable farming in this context is the one that gives the maximum yield, year in and year out. Wheat is grown on more acres worldwide than any other cereal. In the United Kingdom, average organic wheat yields are four tonnes per hectare whilst conventional yields are averaging eight.

I regard the obsession amongst some for organic farming as merely a reflection of wealth, an apparent abundance of food, and a feeling that the problem of food security is solved. But the world for wealthy countries is changing. Global warming, greenhouse gas emissions, biofuels, and soaring oil and food prices are among the many new problems; food security is again becoming a hot topic. Environmental issues, clearly only part of the good times, are declining in relevance.

Although Ronald and Adamchak mention no-till agriculture only briefly, this is surely the agriculture of the future. No-till farms produce only one third of the greenhouse gas emissions of an organic farm [5]. No-till eliminates soil erosion and improves environment, wildlife, and soil. Most importantly, it maintains a conventional yield. Currently 10% of United States farms are totally no-till, and another 60% are partially no-till; this achievement is due almost solely to the availability of GE herbicide-tolerant crops.

No-till is not an amalgam of organic and GE technology but something that was derived from observations of nature in a very different way. Faulkner, the perceptive founder of no-till in 1943 [6], derived his revolutionary ideas from asking himself a very simple question: Why don't the prairies suffer from the present (1940s) problems of US agriculture? Faulkner's answer: the prairies are not subjected to that most damaging of all soil treatments: the plough. Leaving crop residues on the surface is the nearest any form of agriculture comes to mimicking the annual and natural cycle of the meadow. Herbicides are human “allelopathy” of weeds, and humans are part of nature too. If you want an agriculture that is nearest nature, then this is surely it.

**Figure:**
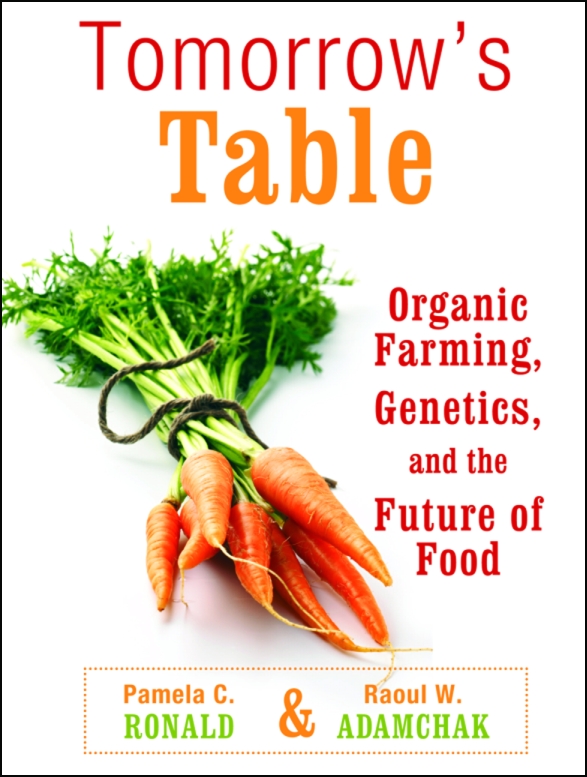


## Abbreviations

GE: genetically engineered
